# Paternal Age in Relation to Offspring Intelligence in the West of Scotland Twenty-07 Prospective Cohort Study

**DOI:** 10.1371/journal.pone.0052112

**Published:** 2012-12-13

**Authors:** Elise Whitley, Ian J. Deary, Geoff Der, G. David Batty, Michaela Benzeval

**Affiliations:** 1 MRC/CSO Social and Public Health Service Unit, Glasgow, United Kingdom; 2 Centre for Cognitive Ageing and Cognitive Epidemiology, Department of Psychology, University of Edinburgh, Edinburgh, United Kingdom; 3 Department of Epidemiology and Public Health, University College London, London, United Kingdom; University of Hong Kong, China

## Abstract

**Background:**

The adverse effects of advancing maternal age on offspring's health and development are well understood. Much less is known about the impact of paternal age.

**Methods:**

We explored paternal age-offspring cognition associations in 772 participants from the West of Scotland Twenty-07 study. Offspring cognitive ability was assessed using Part 1 of the Alice Heim 4 (AH4) test of General Intelligence and by reaction time (RT).

**Results:**

There was no evidence of a parental age association with offspring RT. However, we observed an inverse U-shaped association between paternal age and offspring AH4 score with the lowest scores observed for the youngest and oldest fathers. Adjustment for parental education and socioeconomic status somewhat attenuated this association. Adjustment for number of, particularly older, siblings further reduced the scores of children of younger fathers and appeared to account for the lower offspring scores in the oldest paternal age group.

**Conclusion:**

We observed a paternal age association with AH4 but not RT, a measure of cognition largely independent of social and educational experiences. Factors such as parental education, socioeconomic status and number of, particularly older, siblings may play an important role in accounting for paternal age-AH4 associations. Future studies should include parental intelligence.

## Introduction

Changing trends in education, employment and reproductive technologies have led to a rise in the average age of childbearing in men and women in many industrialised countries since the mid-1970s [1]–[5]. While the adverse effects of advancing maternal age are well understood, [5] considerably less is known about the impact of father's age on the health and development of their offspring. It is known that pregnancies conceived to older men are more likely to end in spontaneous abortion [6] and that their children are at higher risk of birth defects [7]. There is also increasing evidence to suggest that there may be more long-term adverse effects, for example, children born to older fathers appear to have an increased risk autism [8], [9] and schizophrenia [10], [11]. An emerging literature suggests that paternal age may also impact on offspring cognitive abilities [12]–[16]. These studies have all reported lower IQ scores in children with older fathers and all but one [12]–[14], [16] also report lower IQ scores in children of younger fathers. These associations have potentially important consequences as low early-life cognitive ability is associated with subsequent increased mortality [17], [18].

Although an inverse U-shaped association between paternal age and offspring IQ has been reported in several studies, it is not clear what mechanisms may underlie it. Neurobiological hypotheses have been proposed, which may explain all [19] or part of the association; for example, poor offspring outcomes in older fathers may be due to accumulation of chromosomal mutations during male germ cell maturation, [20], [21] a view supported by some animal studies [22]. However, there has also been discussion of the impact of environmental factors such as parental education, socioeconomic status (SES), and family size and position.

It is important to recognise that lower offspring intelligence at the two paternal age extremes may not arise for the same reasons. For example, age at fatherhood is often positively correlated with socioeconomic status (SES), education level and own IQ [23]. This, coupled with inherited intelligence, might explain lower IQ scores among children of younger fathers. Alternatively, it has been suggested that children with younger parents may be at a disadvantage in terms of economic resources and social and cultural capital, [24] and this may impact negatively on their academic performance. However, older fathers have a higher risk of mortality or ill-health and this may have a negative impact on the intellectual environment in the home [13]. In addition, the role of family size and position is complex and worthy of further investigation. Children born to older fathers may have more siblings in general (family size) and more older siblings in particular (family position). Increasing family size is known to be associated with lower IQ [25]. However, the relative importance of family size and family position in explaining paternal age-offspring IQ associations is more equivocal and it has been suggested that family position may explain more of the association than family size [14]. The uncertainty regarding mechanisms means that appropriate adjustment for confounding and mediating variables is essential. The most recent study on paternal age and offspring IQ [26] is a reanalysis of a previously-published dataset, [15] and concludes that previously-reported associations might be a result of under- or missing adjustment for covariates.

In the present report we explore paternal age-offspring cognition associations in a representative sample of West of Scotland residents. Participants' cognition was assessed using a psychometric test of general intelligence and tests of reaction time (RT). RTs are correlated with psychometric intelligence [27]–[30] but, being based on responses to simple stimuli, are less influenced by cultural, educational and social background [31]. Similarities or differences in paternal age associations between RTs and standard intelligence tests may therefore lend more weight to biological or social hypotheses respectively. To our knowledge, paternal age-RT associations have not previously been described. In addition to exploring different measures of cognitive ability, we have also examined a wide range of covariates collected directly from the parents.

## Methods

The West of Scotland Twenty-07 study is a population based multiple-cohort study and has previously been described in detail [32]. Briefly the study comprises three age-cohorts aged around 15, 35, and 55 years at baseline, and followed up for over 20 years. Our analyses are based on the youngest age cohort, for whom parental data were also available. Analyses are based on respondents' data collected at follow-up waves 1 (1987/88), 4 (2000/04) and 5 (2007/08). Data on respondents' parents who were living at home at wave 1 were collected at interview with the parents themselves. Ethical approval for Wave 1 was granted in 1986 by the ethics sub-committee of the West of Scotland Area Medical Committees and the GP Sub-Committee of Greater Glasgow Health Board. Wave 4 was approved by Glasgow University Ethics Committee and Wave 5 was approved by Tayside Committee on Medical Research Ethics A. At each wave written consent was obtained from respondents. At Wave 1 when respondents were aged 15, parental consent was obtained.

Paternal and maternal ages at respondents' birth were calculated for respondents' biological parents, based on respondent and parent age at interview, and are generally accurate to within ±1 year. Cognitive ability was assessed in two ways: (i) based on Part 1 of the Alice Heim 4 (AH4) test of General Intelligence, and (ii) based on RTs. AH4 has been used widely in cohort studies in the UK as a reliable and valid measure of general mental ability [33]. The test is based on 65 items, including verbal and numerical reasoning, of which the participant completes as many as possible in ten minutes. Administration and scoring were carried out according to instructions in the test manual [33] and a practise test was given before the test proper [33]. The current analyses are based on AH4 measured at the 5^th^ wave (when respondents were aged approximately 35) or, if AH4 was missing at that interview, from the 4^th^ wave (aged approximately 28). Among respondents with complete data, AH4 scores in waves 4 and 5 were highly correlated (correlation coefficient = 0.86 p<0.001), which supports this use of 4^th^ wave data for some participants. Simple and four-choice RTs were measured with a portable device designed for the UK Health and Lifestyle Survey, [34] and have been previously described in detail [27]. The same reaction time device has also been used in the large, population-representative Health and Lifestyle Study in the UK, [29] and in the Lothian Birth Cohort Studies [28]. In the simple RT test, the participant rested the second finger of their preferred hand on a key marked 0 and pressed this key as quickly as possible when a zero appeared on an LCD screen. There were eight practise tests and 20 test trials, and an inter-stimulus interval that varied between 1 and 3 seconds, and the mean and standard deviation (SD) of the 20 test trials was calculated for each participant. In the four-choice RT test, the participant rested the second and third fingers of the left and right hands on keys marked 1, 2, 3, and 4 respectively. When a number (between 1 and 4) appeared on the LCD screen the participant attempted to press the correct key as quickly as possible. There were eight practise trials and 40 test trials, and an inter-stimulus interval that varied between 1 and 3 seconds, and the mean and SD of correct and incorrect trials were calculated separately. The current analyses are based on the mean of the correct trials and, as described previously, [35] participants with 10 or more incorrect responses were not included. RTs were measured at age 15 (wave 1) and again at approximately 35 years (wave 5).

Previous evidence [26] highlights the importance of adjusting paternal age-offspring IQ associations for potentially confounding or mediating variables. We therefore explored the impact of a wide range of factors that may be associated with both paternal age and offspring cognition. These data were collected directly from parents at wave 1. Retrospective questions were asked regarding the respondent and also the occupational SES of both parents at the respondent's birth. Other factors, including parental health, parental behaviours, parental attitudes, respondent health, family size and position, were asked when the respondent was aged 15. We therefore primarily consider these factors as potential mediating variables. However, the parental variables measured at respondent age 15 are likely to be correlated with the same variables at or before the respondent's birth and we therefore cannot rule out the possibility that they also have a confounding effect on paternal age-offspring IQ associations. We discuss the role of these factors in the context of specific hypothesised mechanisms in the Discussion section. Parental variables included in the current analyses were, for both parents: occupational SES at respondent's birth (IV/V, IIIM, IIINM, I/II); and, at respondent age 15, highest educational qualification (none, school, further/higher education); household income (quartiles); long-standing illness or chronic disease (any vs. none); smoking status (current vs. not); drinking status (regular drinker vs. not); and participation in sport (regular vs. not). We also included variables designed to assess parental attitudes to work, education and autonomy at respondent age 15 (agreement vs. disagreement with statements: “I have very little control over my life”, “Success in life is largely a matter of hard work”, “If you're determined it is possible to get a job”, “School subjects useless for jobs should be scrapped”, and “It is important that my child does well at school”). Offspring variables, also collected from parents at wave 1, were: whether there were any pregnancy or birth complications; birthweight; whether child was breastfed; whether child has any long-standing illness or disability at age 15; how often child eats with the rest of the family at age 15; and the number of all, older, and younger siblings at age 15.

Paternal age-offspring cognition associations were explored using least squares regression and likelihood ratio tests. Given previously reported inverse U-shaped associations, preliminary analyses treated paternal age as a continuous variable and included linear and quadratic terms. The addition of higher order terms did not improve the fit of the model, based on conventional levels of statistical significance. For illustration, we also present results for paternal age in four categories. All analyses are adjusted for age at cognitive assessment. Models adjusted for covariates were built parsimoniously, based on a priori inclusion of potentially important variables, and the strength of bivariate associations. Results are based on respondents with complete data on paternal age, AH4, RTs, and covariates of interest. For comparison, we also explored maternal age-offspring cognition associations in the same way.

## Results

A total of 2,539 individuals aged 15 were initially approached to take part in the study and 1,515 (59.7%) agreed to participate. The baseline sample has been shown to be representative of the general population in the study area [36]. Of these, 199 (13.1%) had missing paternal age, 491 (32.4%) missing AH4 scores or RTs, and 53 (3.5%) missing data for at least one covariate, leaving a total of 772 (51.0%) in our analytical sample. Excluded respondents tended to have less educated and lower SES parents. Respondents with data on paternal age had slightly higher AH4 scores (mean (standard deviation (SD)) AH4 in respondents included vs. excluded from analyses: 39.3 (10.0) vs. 37.7 (10.4)) and lower RTs (536.0 (66.3) vs. 545.7 (78.2) milliseconds) than those without; those with cognitive measures were almost identical to those without, in terms of paternal age (29.9 (5.9) vs. 29.6 (6.2) years).

Characteristics of respondents and their parents by paternal age at respondent birth are shown in Table 1. Maternal age clearly increased with increasing paternal age, as would be expected due to assortative mating, although mothers tended to be around two years younger than fathers at the time of their child's birth. Relative to those with the youngest fathers, respondents with older fathers tended to have parents with higher SES at the time of their birth, with the exception of the oldest paternal age group where parental SES was lower. Similar, but weaker patterns were observed for parental education and income. Paternal age associations with parental health were inconsistent and generally weak. There was a scant suggestion of more long-standing illness in older fathers, and weak evidence that older parents might smoke and drink less. There was little or no evidence of changing attitudes to work, education and autonomy with increasing paternal age. The offspring of older fathers weighed increasingly more at birth, but pregnancy/birth complications, breastfeeding, offspring illness, and eating as a family were largely independent of paternal age. In contrast, there were strong associations between paternal age and family size and position. Respondents with older fathers tended to have more siblings in total but there was also a clear difference in the numbers of older and younger siblings by paternal age, with the offspring of older fathers having increasing numbers of older and decreasing numbers of younger siblings when compared with those with younger fathers. Finally, mean AH4 score was lowest among respondents with the youngest fathers, rising in those with fathers aged 25–29 and 30–34, before falling again in respondents with the oldest fathers. In contrast, mean four-choice RT in wave 5 was similar in all paternal age groups and this was also the case for simple RT in wave 5 and both RTs in wave 1 (results not shown).

**Table 1:** Offspring and parental characteristics by paternal age at offspring birth.

	Paternal age			
	<25	25–29	30–34	35+
N	144	254	217	157
**Parental characteristics**				
Mean (SD) maternal age^1^	21.8 (2.2)	25.4 (2.6)	29.6 (3.1)	34.0 (4.8)^***^
% SES I or II (father)^1^	9.7	20.9	32.7	25.5^***^
% SES I or II (mother)^1^	14.3	15.5	26.5	18.6^***^
% higher education (father)^2^	27.8	33.9	42.9	33.8
% higher education (mother)^2^	19.2	27.5	34.7	21.8^*^
% household income highest quartile^2^	15.0	26.3	32.9	20.6^**^
% no long standing illness (father)^2^	80.6	79.1	74.2	70.7
% no long standing illness (mother)^2^	77.1	79.5	82.5	79.0
% non-smoker (father)^2^	51.1	52.8	56.7	56.1
% non-smoker(mother)^2^	57.7	50.1	67.8	67.3^**^
% not a heavy drinker (father)^2^	57.3	46.2	51.2	60.7^*^
% not a heavy drinker (mother)^2^	69.5	68.8	70.7	80.8^*^
% regular sports participation (father)^2^	36.4	32.7	30.2	25.3
% regular sports participation (mother)^2^	30.5	24.5	29.3	19.4
% have control over life (father)^2^	78.3	81.5	73.4	78.1
% have control over life (mother)^2^	79.0	86.6	85.9	80.0
% hard work = success (father)^2^	67.8	70.0	71.7	64.1
% hard work = success (mother)^2^	66.4	76.4	74.0	68.4
% can get a job (father)^2^	47.1	50.7	51.4	51.2
% can get a job (mother)^2^	48.4	55.2	53.7	50.4
% keep non-vocational subjects (father)^2^	53.7	59.5	65.6	55.0
% keep non-vocational subjects (mother)^2^	50.0	68.9	67.9	61.8^**^
% school success very important (parent)^2^	55.6	54.5	54.5	58.1
**Offspring characteristics**				
Mean (SD) birthweight (kg)	3.2 (0.6)	3.3 (0.6)	3.4 (0.5)	3.4 (0.6)^ **^
% no pregnancy/birth complications	63.6	69.9	65.1	69.1
% breastfed	13.9	20.1	22.2	16.7
% no long standing illness^2^	69.4	75.6	75.6	75.8
% eat with family daily^2^	85.3	89.0	86.6	84.7
Mean (SD) total number of siblings^2^	1.7 (1.0)	1.8 (1.2)	2.0 (1.4)	2.8 (1.7)^ ***^
Mean (SD) number of older siblings^2^	0.4 (0.7)	0.9 (1.1)	1.4 (1.2)	2.5 (1.7)^ ***^
Mean (SD) number of younger siblings^2^	1.3 (1.0)	0.9 (0.9)	0.6 (0.9)	0.4 (0.6)^ ***^
Mean (SD) AH4 score (age 28 or 35)	37.1 (10.2)	39.3 (9.7)	41.4 (10.2)	38.4 (9.4)^ *^
Mean (SD) choice RT^3^ (age 35)	541 (71)	540 (68)	529 (64)	536 (63)

1 At offspring birth;^ 2^At offspring age 15; ^3^Mean four-choice reaction time in milliseconds based on correct responses only; ^***^p for heterogeneity across categories <0.001; ^**^p for heterogeneity across categories <0.01;^ *^p for heterogeneity across categories <0.05.

**Figure 1:**
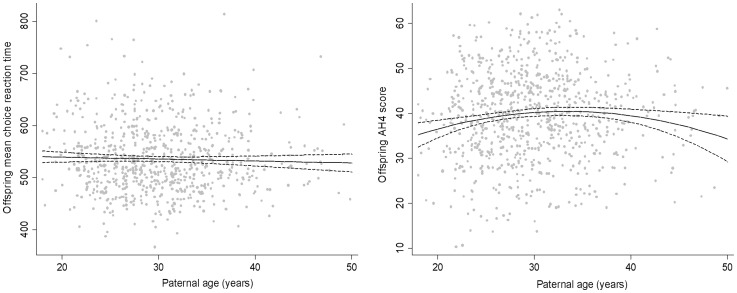
Offspring mean choice reaction time^1^ (at approximate age 35) and AH4 score^2^ (at approximate age 28 or 35) by father '**s age (with 95% confidence intervals).** ^1^Mean (standard deviation) mean choice reaction time (based on correct responses in those with <10 incorrect responses): 536.0 (66.3) milliseconds; ^2^Mean (standard deviation) AH4 score: 39.3 (10.0).

Paternal age associations with cognitive ability, based on regression models with paternal age included as a continuous variable, were very similar to the descriptive categorical results. There was no evidence of a paternal age association (linear or quadratic) with mean simple or choice RTs at either age (for example Figure 1a). In contrast there was evidence (p = 0.002) of a quadratic paternal age association with AH4 (Figure 1b); respondents with the youngest and oldest fathers tended to have lower AH4 scores. Regression coefficients (linear and quadratic) for paternal and maternal age associations with AH4 scores and RTs are shown in Table 2. AH4 associations with maternal age were weaker than those for paternal age and were fully attenuated by adjustment for paternal age. In contrast, paternal age-AH4 associations were only marginally attenuated by adjustment for maternal age. There was no evidence that either paternal or maternal age was associated with mean simple or four-choice RT at either age (Table 2 presents results for choice reaction time in wave 5).

**Table 2:** Regression coefficients (95% confidence intervals) for AH4 score (age 28 or 35) and mean choice reaction time (age 35) according to father's and mother's age (in years) at respondent's birth.

	Adjusted for age at IQ measurement	Adjusted for age at IQ measurement and other parent's age at birth
AH4 score		
Paternal age (linear term)	1.57 (0.65, 2.50)	1.48 (0.18, 2.77)
Paternal age (quadratic term)	−0.02 (−0.04, −0.01)	−0.02 (−0.04, −0.01)
* P* 1	*0.002*	*0.02*
Maternal age (linear term)	1.09 (−0.01, 2.18)	−0.07 (−1.58, 1.44)
Maternal age (quadratic term)	−0.02 (−0.04, 0.00)	0.01 (−0.02, 0.03)
* P* 1	*0.02*	*0.19*
Reaction time		
Paternal age (linear term)	−5.40 (−11.91, 1.10)	−3.92 (−13.03, 5.20)
Paternal age (quadratic term)	0.08 (−0.02, 0.18)	0.05 (−0.09, 0.19)
* P* 1	*0.21*	*0.63*
Maternal age (linear term)	−5.92 (−12.98, 1.14)	−2.75 (−13.31, 7.81)
Maternal age (quadratic term)	0.10 (−0.02, 0.22)	0.05 (−0.12, 0.23)
* P* 1	*0.21*	*0.77*

1 p for model including both linear and quadratic terms based on likelihood ratio test.

Differences in mean AH4 by categories of paternal age are shown in Table 3. The highest mean AH4 score was observed among respondents with fathers aged 30–34 at the time of their birth. The lowest was among respondents with the youngest and, to a lesser extent, oldest fathers. Adjustments for parental income, health, health behaviours, attitudes to work, education and autonomy, and respondent birthweight, breastfeeding, health, and long-standing illness had no marked impact on these associations. Adjustment for father's education and, more markedly, SES somewhat reduced the estimated difference in all but the oldest paternal age group; adjustments for mother's education and SES had a similar or weaker impact (results not shown). Adjusting for number of siblings had a complex effect, accentuating differences at younger paternal ages but, if anything, suggesting *higher* offspring AH4 scores in the 35+ paternal age group compared with the <30 years age groups, although the confidence intervals were wide. This effect was strongest after adjustment for number of older siblings; there was no impact of adjustment for number of younger siblings. After simultaneous adjustment for factors having the greatest impact, namely number of older siblings, paternal education and paternal SES, a similar, slightly weaker pattern of increasing offspring AH4 with increasing paternal age was observed; a quadratic term no longer improved the fit of this model.

**Table 3:** Difference (95% confidence interval) in mean AH4 score according to father's age at respondent's birth.

	N	Adjusted for age at IQ measurement	Adjusted for age and father's education^1^	Adjusted for age and father's SES^1^	Adjusted for age and total number of siblings	Adjusted for age and number of older siblings	Multiply adjusted^2^
All respondents (N = 772)							
<25	144	−2.15 (−4.17, −0.14)	−1.96 (−3.91, −0.01)	−1.15 (−3.10, 0.80)	−2.25 (−4.24, −0.26)	−3.08 (−5.07, −1.08)	−2.11 (−4.04, −0.17)
25–29	254	0.00 (ref)	0.00 (ref)	0.00 (ref)	0.00 (ref)	0.00 (ref)	0.00 (ref)
30–34	217	1.98 (0.19, 3.77)	1.47 (−0.26, 3.21)	1.25 (−0.48, 2.97)	2.23 (0.47, 4.00)	2.74 (0.97, 4.50)	1.74 (0.03, 3.45)
35+	157	−0.94 (−2.90, 1.02)	−1.00 (−2.90, 0.90)	−1.09 (−2.97, 0.80)	0.31 (−1.69, 2.31)	1.65 (−0.45, 3.75)	0.88 (−1.14, 2.89)
* P^3^*		*<0.001*	*0.01*	*0.05*	*<0.001*	*<0.001*	*0.004*

1 Adjustment for mother's education and SES had a similar or weaker impact on these associations; ^2^Adjusted for age, father's education, father's SES, and number of older siblings; ^3^p for heterogeneity based on likelihood ratio test.

We also explored the joint effects of number of older siblings and parental education and SES, by stratifying analyses by the number of older siblings. We present paternal education- and SES-adjusted results for respondents with no older siblings, one older sibling, and two or more older siblings in Table 4 (p for interaction between paternal age and no vs. 1+ older siblings  = 0.06). These analyses are based on relatively small numbers of individuals, meaning that confidence intervals around estimates are inevitably wide and results should therefore be interpreted with caution. Among respondents with no older siblings, mean AH4 score increased with advancing paternal age, particularly after adjustment for paternal SES, and the highest offspring AH4 scores were observed in the oldest paternal age group. Results for respondents with one or more older siblings were less marked. However, while there was still evidence of lower AH4 scores in respondents with the youngest fathers, there was little or no evidence of a decrease in AH4 scores amongst those with the oldest fathers in these stratified analyses.

**Table 4:** Difference (95% confidence interval) in mean AH4 score according to father's age at respondent's birth stratified by number of older siblings.

	N	Adjusted for age at IQ measurement	Adjusted for age and father's education^1^	Adjusted for age and father's SES^1^	Multiply adjusted^2^
Respondents with no older siblings (N = 283)					
<25	102	−2.41 (−5.03, 0.22)	−2.07 (−4.66, 0.52)	−1.45 (−3.97, 1.08)	−1.49 (−4.01, 1.03)
25–29	107	0.00 (ref)	0.00 (ref)	0.00 (ref)	0.00 (ref)
30–34	55	5.00 (1.84, 8.16)	4.85 (1.74, 7.97)	4.05 (1.02, 7.07)	4.20 (1.17, 7.22)
35+	19	5.16 (0.42, 9.90)	5.00 (0.34, 9.66)	6.25 (1.73, 10.77)	6.08 (1.57, 10.60)
* P^3^*		*<0.001*	*<0.001*	*<0.001*	*<0.001*
Respondents with one older sibling (N = 233)					
<25	32	−4.45 (−8.48, −0.43)	−3.50 (−7.35, 0.34)	−2.26 (−6.16, 1.63)	−2.23 (−6.06, 1.60)
25–29	88	0.00 (ref)	0.00 (ref)	0.00 (ref)	0.00 (ref)
30–34	81	1.37 (−1.63, 4.37)	0.78 (−2.06, 3.63)	0.58 (−2.32, 3.47)	0.40 (−2.43, 3.24)
35+	32	0.55 (−3.47, 4.57)	0.07 (−3.74, 3.88)	0.03 (−3.82, 3.87)	−0.11 (−3.89, 3.65)
* P^3^*		*0.04*	*0.18*	*0.56*	*0.60*
Respondents with two or more older siblings (N = 256)					
<25	10	−3.36 (−9.64, 2.93)	−3.57 (−9.72, 2.58)	−2.81 (−9.05, 3.44)	−3.13 (−9.31, 3.05)
25–29	59	0.00 (ref)	0.00 (ref)	0.00 (ref)	0.00 (ref)
30–34	81	2.21 (−0.95, 5.36)	1.26 (−1.87, 4.38)	1.71 (−1.43, 4.86)	1.11 (−2.02, 4.25)
35+	106	0.45 (−2.53, 3.42)	−0.04 (−2.96, 2.89)	−0.11 (−3.08, 2.86)	−0.29 (−3.23, 2.65)
* P^3^*		*0.21*	*0.40*	*0.34*	*0.48*

1 Adjustment for mother's education and SES had a similar or weaker impact on these associations; ^2^Adjusted for age, father's education, and father's SES; ^3^p for heterogeneity based on likelihood ratio test.

## Discussion

Previous reports suggest that children born to younger [12]–[14], [16] and older [12]–[16] fathers have lower IQ scores. Discussions in the literature have considered biological mechanisms; for example, poorer offspring cognition in older fathers might be a result of increasing male germ cell mutations, [20]–[22] and also environmental factors such as parental SES and family size and position. We observed an inverse U-shaped age-adjusted association between paternal age and offspring AH4 score. However, if there was a biological basis for these associations, we would expect similar or stronger paternal age associations with RTs, a measure of cognition that is largely independent of social and educational experiences [31]. In contrast, we found no paternal age-RT association. We are unaware of any other studies that examine RTs.

Results from studies in which adjustments were made [13]–[15] suggest that factors such as parental education, paternal SES, and number of siblings have an attenuating effect on associations of father's age with offspring IQ. The most recent study [26] was a re-analysis of existing data and reported that previously-observed associations [15] were markedly attenuated by additional adjustment for maternal education and number of siblings, concluding that previously-reported associations may be due to under- or missing adjustment for such factors. In the current analysis, detailed information was collected directly from parents and we were therefore able to explore a wide range of covariates, including the majority of those included in previous studies. These cover factors relating to parental SES, education, health, and health behaviours, as well as attitudes to education and employment which have not been previously explored to our knowledge. Data collected from parents also allowed us to directly examine a range of factors relating to the pregnancy and subsequent health of the respondent, in addition to detailed information on the number and age of any siblings. As stated previously, the majority of our covariates were based on the respondent's and parents' status at respondent age 15; the exception was retrospective ascertainment of the respondent's birth characteristics and parents' SES at the time of the respondent's birth. We therefore primarily considered covariates as mediating variables in our analyses; however, we cannot rule out confounding effects by these same covariates as there is likely to be a correlation between parental characteristics at respondent age 15 and the same characteristics at or before the respondent's birth.

The majority of adjustments for covariates had little or no impact on paternal age-AH4 associations. However, in common with previous studies, associations in all but the oldest paternal age group were attenuated by adjustment for parental education and SES, particularly those of the father. It is generally reported that older parents tend to be better educated and to have a higher SES, making the tendency towards lower IQ scores in the offspring of older men somewhat unexpected. In our data, parental SES and education increased with father's age up to 35 and then fell again. Given the similarities of these associations and those with offspring IQ, it is not surprising that we observed some attenuation in paternal age-offspring IQ associations after adjustment for paternal education and SES, although these adjustments did not wholly explain the association. However, it is important to note that respondents in our cohort were all born in 1971/72, meaning that parents who were older at the respondent's birth were themselves from earlier birth cohorts and were therefore educated in different circumstances to those who were younger, e.g. when the legal school leaving age was lower. As a result, our measures of parental education and SES may be more strongly influenced by external factors, e.g. temporal trends and social circumstances in childhood, than by IQ. So although parental SES and education seems to mirror offspring IQ this may not accurately reflect parental intelligence per se. These results highlight the need for future studies to include a direct measure of parental IQ.

In common with previous studies, we also found that number of siblings had an impact on paternal age-offspring IQ associations. Although family size (total number of siblings) is more strongly associated with IQ than family position (number of older siblings), [25] we have replicated a previous result, [14] suggesting that family position has a stronger confounding effect on paternal age-offspring IQ associations. This result requires further confirmation but may reflect a stronger paternal age association with number of, specifically, older siblings. It has been mooted that the negative impact on IQ of increasing numbers of siblings may be a consequence of dilution of parental economic, social and emotional resources, leading to reduced stimulation and attention, and therefore cognition [37]. There is debate regarding this hypothesis but it is consistent with our results and might explain the apparent decrease in offspring cognition at older paternal ages.

There are a number of limitations to our analyses. The sample size was relatively modest, resulting in generally wide confidence intervals, particularly in the analyses restricted to respondents with no older siblings, which limited the potential to explore the oldest paternal ages in detail. Parental ages were accurate to within ±1 year and, although we have no reason to suppose this will have biased our results, it may have weakened them somewhat. In common with previous studies, we found that parental education and, particularly, SES had a role in explaining paternal age-offspring IQ associations. However, we are not able to establish what aspects of parental SES influence offspring IQ. It is possible that children with older parents may benefit cognitively as a result of greater economic resources and social and cultural capital [24]. It is also likely that parents' SES is influenced by their intelligence, which is inherited by their offspring. However, as discussed above, other factors will also be involved and, in common with existing studies, although we had extensive data on many related factors, including both parents' SES and education, it is likely that we not have adequately accounted for the role of parental IQ.

Finally, our analyses were based on just over 50% of the original sample, after excluding those with missing data. Only parents who were living with the respondent at wave 1 were interviewed and so divorced or separated parents who had left the family home were not included. Given the tendency, particularly in the 1980s, for mothers to gain custody of their children, this is likely to have led to a greater loss of data relating to fathers, including paternal age, and it is possible that there are differences between respondents included and excluded from our analyses. Excluded respondents tended to have less educated or lower SES parents, who might also be expected to have had lower IQ scores. This would be consistent with the observation that respondents who were excluded because of unknown paternal age tended to have slightly lower AH4 scores and higher RTs than those with complete paternal age information. These exclusions may have affected our results in three ways. The first is in terms of generalizability and our results may be more relevant to individuals with higher SES parents. The second is that we may have underestimated the impact of parental SES and education on explaining parental age-offspring IQ associations. The third is the possibility that our results arise as a result of selection bias and we cannot categorically rule out this possibility. However, if this is the underlying explanation then this would imply that we under-sampled respondents with lower SES fathers specifically in the two middle age ranges (i.e. aged 25–34). We have no reason to suppose that this is the case and, indeed, the paternal age distribution of respondents excluded because of missing cognitive data was almost identical to that of respondents included in our analyses. In addition, our results are consistent with other studies that have explored similar associations in a range of populations and it is unlikely that these are all a result of selection bias.

### Conclusion

Low cognitive ability impacts on later education and SES, and is also associated with increased mortality. Our results suggest that father's age at birth may be associated with offspring AH4 but not RTs and that this association may be due, at least in part, to parental education, parental SES, and number of, particularly older, siblings. The impact of these inter-related factors and, most importantly, parental IQ is complex and worthy of further exploration. Future studies should include directly measured parental IQ. In addition, separate follow-up of children whose fathers have and have not left the family home will help to disentangle biological and environmental mechanisms, and a better knowledge of the reasons for early and late fatherhood will also enhance our understanding of the mechanisms underlying paternal age-offspring cognition associations. A greater understanding of the impact of both mother's and father's age on offspring health and development will highlight some of the consequences for society of the increasing demographic trend in western societies for couples to begin families at older ages. However, with respect to how individuals might regard these findings for their own situation, we should stress that these are relatively small associations found in a substantial sample.
